# Optical coherence tomography and color fundus photography in the screening of age-related macular degeneration: A comparative, population-based study

**DOI:** 10.1371/journal.pone.0237352

**Published:** 2020-08-14

**Authors:** Edoardo Midena, Luisa Frizziero, Tommaso Torresin, Paolo Boscolo Todaro, Giacomo Miglionico, Elisabetta Pilotto

**Affiliations:** 1 Department of Ophthalmology, University of Padova, Padova, Italy; 2 IRCCS–Fondazione Bietti, Rome, Italy; University of Florida, UNITED STATES

## Abstract

**Purpose:**

To analyze the individual value and the contribution of color fundus photography (CFP) and optical coherence tomography (OCT) in the screening of age-related macular degeneration (AMD) of an unselected population.

**Methods:**

CFP and OCT images of 15957 eyes of 8069 subjects older than 55 years, obtained during a population-based screening for AMD using a single diagnostic non-mydriatic imaging device, were analyzed by a blinded examiner. The two techniques were preliminary evaluated considering the dichotomous parameter "gradable/ungradable", then gradable images were classified. CFP were graded according to the standardized classification of AMD lesions. OCT images were also categorized considering the presence of signs of early/intermediate AMD, late AMD, or other retinal diseases. Another blinded operator re-graded 1978 randomly selected images (for both CFP and OCT), to assess test reproducibility.

**Results:**

Of the 15957 eyes, 8356 CFP (52.4%) and 15594 (97.7%) OCT scans were gradable. Moreover, most of the eyes with ungradable CFP (7339, 96.6%) were gradable at OCT. AMD signs were revealed in 7.4% of gradable CFP and in 10.4% of gradable OCT images. Moreover, at OCT, AMD signs were found in 1110 (6.9%) eyes whose CFP were ungradable or without AMD (847 and 263 eyes, respectively). The inter-operator agreement was good for the gradable versus ungradable parameter, and optimal for the AMD grading parameter of CFP. The agreement was optimal for all OCT parameters.

**Conclusions:**

OCT provided gradable images in almost all examined eyes, compared to limited CFP efficiency. Moreover, OCT images allowed to detect more AMD eyes compared to gradable photos. OCT imaging appears to significantly improve the power of AMD screening in a general, unselected population, compared to CFP alone.

## Introduction

Age-related macular degeneration (AMD) is the most common cause of visual impairment and blindness in developed countries, particularly in people older than 55 years [1,2]. A worldwide prevalence of 8.7% has been reported, increasing to 17.6% in people older than 70 and to 25% in people older than 80 years [1,2]. Moreover, the estimates for people with European ancestry resulted higher compared with the global estimates and these numbers are expected to increase to 15% by 2050, mainly because of population ageing [2].

In the early phases of AMD visual symptoms may be absent or mild, thus unnoticed by the patient. Conversely, the late phases of AMD (i.e. geographic atrophy and neovascular AMD) are characterized by a significant central visual loss, causing a relevant limitation of daily life activities. The introduction of anti-vascular endothelial growth factor (VEGF) intravitreal therapy has notably improved the visual prognosis for patients affected by neovascular AMD, mainly providing a limitation of macular lesions and the maintenance of a relatively good visual acuity [3,4]. However, patients often refer to physician when the disease has already caused significant and irreversible macular damage and vision loss. Therefore, the early identification of patients at risk is extremely important [5]. Nevertheless, we still lack an efficient screening program because of the absence of recognized, sensible diagnostic tools and a standardized follow-up program [1,6]. Color fundus photography (CFP) is currently the gold standard for AMD screening and allows the detection and grading of this disease [4,7,8]. However, CFP analysis may be sometimes difficult because of poor image quality (media opacities, pupil size and other technical conditions, such as technical ophthalmic imaging expertise, in particular) limiting the grading of AMD lesions [7,9].

A more efficient diagnostic protocol might significantly modify our attitude toward a widespread screening program allowing the early and correct diagnosis and management of this disease. Moreover, some studies have been planned to test new drugs for both treating or slowing down the progression of dry AMD [10]. Along with the progression of the therapeutic research, the application of a verified screening program may allow the early detection of treatable AMD lesions.

Optical coherence tomography (OCT) is one of the most recently developed retinal imaging modalities, providing a high-quality, cross-sectional analysis of the retina, retinal pigment epithelium, and choroid with depth-resolved segmentation and histology-like resolution. OCT has become widely used in the clinical management of retinal diseases, such as AMD, as well as in research clinical trials to assess the fine morphologic retinal changes, both quantitatively and qualitatively [7].

The aim of this study was to analyze the contribution of OCT added to CFP in the screening of AMD in a general, unselected population.

## Methods

This was an observational study approved by the local institutional ethics committee (Ethics Committee for Clinical Practice of the Azienda Ospedaliera di Padova. Number: Prot 2522P/AOP/2014) and following the tenets of the Declaration of Helsinki. All CFP and OCT images collected during a population-based screening for AMD, conducted between 2014 and 2015 under the patronage of the Italian Section of the International Agency for the Prevention of Blindness (IAPB), were retrospectively analyzed. The screening involved a total of 8069 subjects older than 55 years, who voluntarily underwent fundus photography and Spectral Domain OCT examination. Both investigations were simultaneously obtained with a single non-mydriatic device (Topcon 3D OCT-1 Maestro, Topcon Europe Medical BV. Capelle an den IJssel. The Netherlands, version 8.20), on a hi-tech mobile unit. A single 5 figures identification code (ID) was assigned to each subject. A single fifty degrees non-mydriatic CFP centered onto the fovea was obtained for each eye, and then analyzed. The OCT analysis was performed on a single horizontal, 6 mm linear scan with fixation center onto the fovea, in both eyes. A non-sequential analysis of all images of the 8069 subjects was performed by one grader (operator 1), for a total of 15957 images for both fundus photos and OCT scans, respectively. All CFP and OCT images were anonymously and independently analyzed to prevent the influence of one imaging analysis on the other. Moreover, the both CFP and OCT images of 1978 eyes from 1000 randomly selected patients (22 contributing with only one eye) were separately graded by another blinded operator, to assess test reproducibility (operator 2).

### Database and images grading

**Fig 1:**
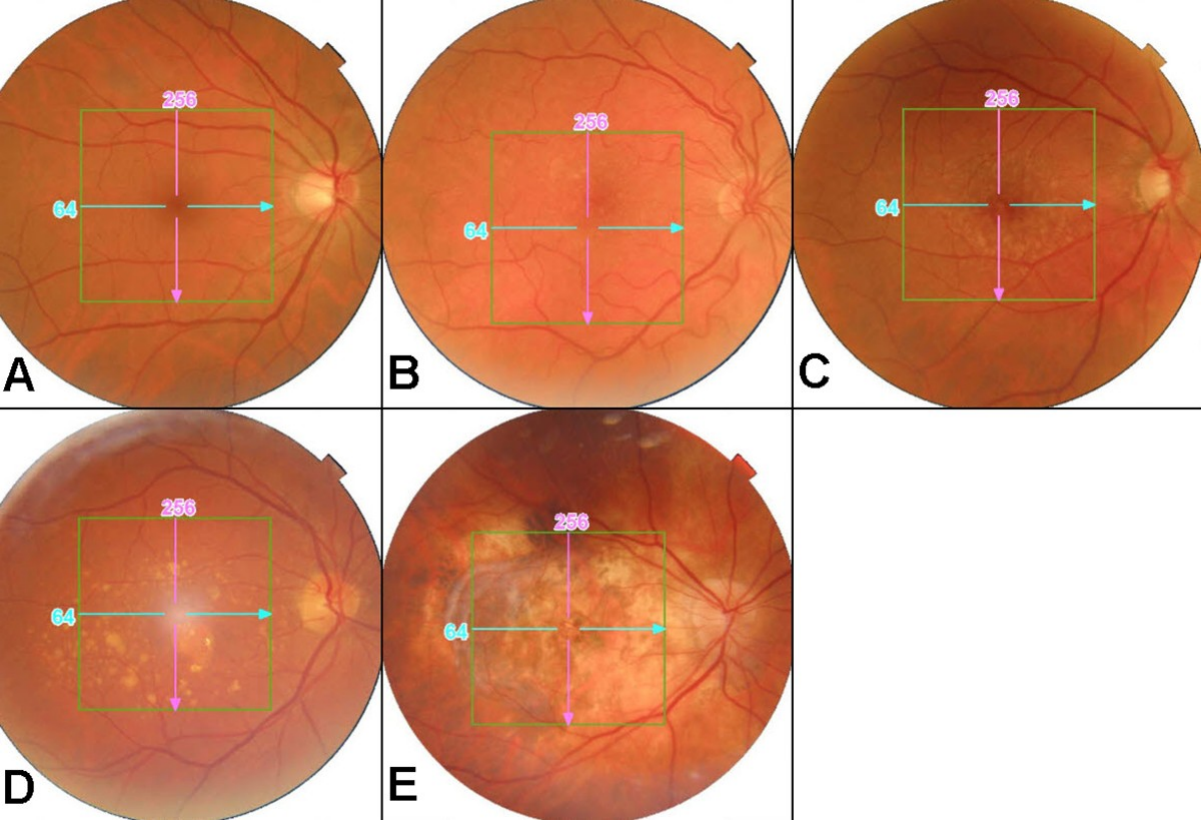
Fundus photo grading. Examples of fundus photos grading: photos graded as score 0 (A), score 1 (B), score 2 (C), score 3 (D) and score 4 (E).

**Fig 2:**
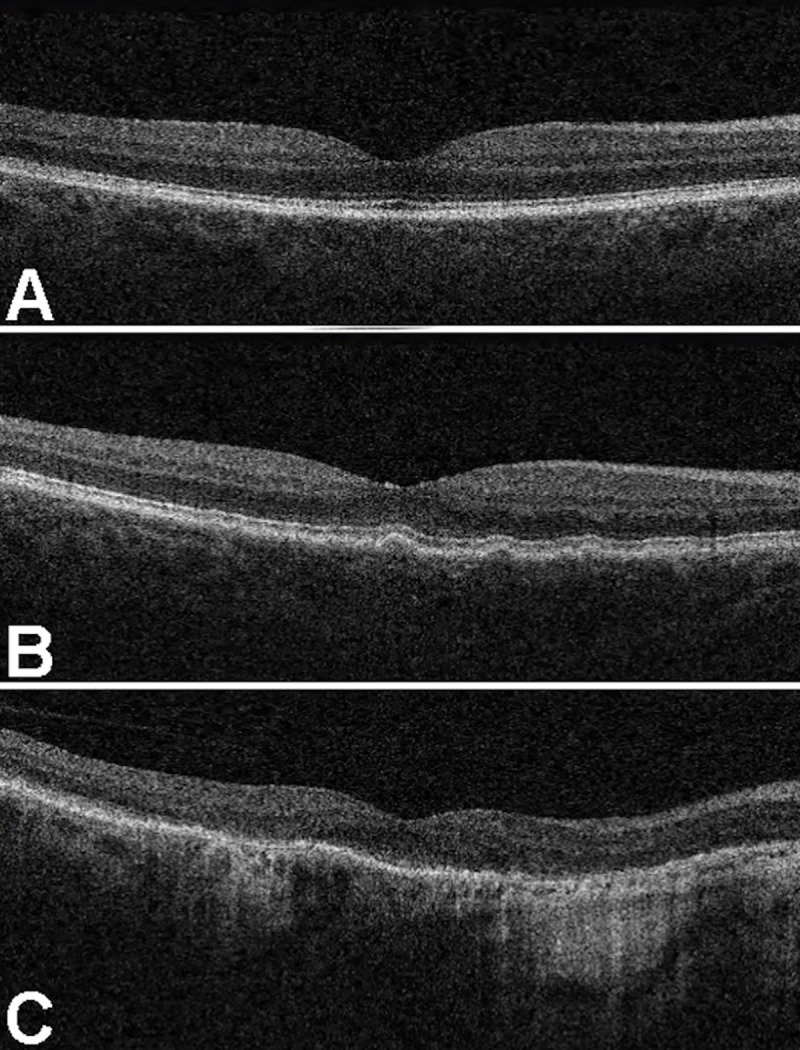
Optical coherence tomography grading. Examples of optical coherence tomography scans grading: images graded as score 0 (A), score 1 (B) and score 2 (C).

For each image the dichotomous parameter "gradable/ungradable" was firstly recorded, for both CFP and OCT scan separately. Then, grading was separately performed for CFP and OCT scans, for each gradable image. CFP were graded according to the Clinical Classification System: score 0 was assigned to normal (no abnormality) findings, 1 to normal ageing changes, 2 to early AMD (i.e. medium size drusen), 3 to intermediate AMD (i.e. large drusen and/or pigmentary changes) and 4 to late AMD (i.e. neovascular AMD signs and geographic atrophy) (Fig 1) [4]. Moreover, score 5 was assigned in case of detection of other retinal diseases except AMD. OCT gradable images were categorized according to the following OCT score: 0 for no abnormal findings, 1 for chorioretinal changes related to early/intermediate AMD (i.e. retinal pigment epithelium abnormalities, drusen), 2 for late AMD (i.e. neovascular AMD signs and geographic atrophy) and 3 for other retinal diseases, except AMD (Fig 2) [11–15].

### Statistical analysis

The evaluation of CFP and OCT scans analysis performed by the operator 1 and the comparison between CFP and OCT scans were performed in terms of similarities and differences, strengths and weaknesses of one compared to the other. This analysis was performed using predominantly descriptive methods: absolute and relative frequency tables (percentage) for the descriptions of the individual examination types and contingency tables for the comparison between examination types. The validation of the analysis was performed on images evaluated by operators 1 and 2, and was carried out using contingency tables and the coefficient of agreement (AC1) according to Gwet, and its 95% confidence interval [16]. The inter-operator agreement of CFP concerned: gradable versus ungradable photos, presence or absence of AMD and AMD grading. For OCT images the same evaluation was performed considering the above mentioned score. Interpretation of agreement was graded as: 0–0.3 = no agreement, 0.3–0.5 = weak agreement, 0.5–0.6 = moderate agreement, 0.6–0.8 = good agreement, 0.8–1.0 = strong agreement.

## Results

CFP and OCT images of 15957 eyes were randomly and independently evaluated by operator 1. Among these, 8356 CFP (52.4%) and 15594 OCT images (97.7%) were gradable. Gradable OCT images were obtained in 96.6% of eyes (7339) with ungradable CFP, and in 98.8% (8255) of eyes with gradable CFP. The main reasons for ungradable CFP (7601) were: poor quality due to media opacities and eye movements artifacts in 2303 (30.3%); defocus in 2235 (29, 4%); insufficient pupil size in 2113 (27.8%); poor field definition in 950 (12.5%). The main reasons for ungradable OCT images (363) were: poor signal due to media opacities and eye movements artifacts in 210 (58%) and lack of foveal centration in 153 (42%).

Among gradable CFP, no signs of AMD (i.e. grade 0 and 1) were detected in 6839 (81.8%) eyes: 6281 and 558 for grade 0 and grade 1, respectively. AMD was detected in 617 (7.4%) eyes: 3.9% early AMD (grade 2), 2.3% intermediate AMD (grade 3) and 1.2% late AMD (grade 4). In 10.8% other retinal diseases were detected (900 images, grade 5).

Among gradable OCT images, no signs of AMD (i.e. grade 0) were detected in 12622 (80.9%) eyes. AMD was detected in 1615 (10.4%) OCT images, including 282 (1.8%) late AMD (grade 2). In 8.7% eyes other retinal diseases were detected (1357 images, grade 3).

The comparison between the two imaging modalities is reported in Table 1. In particular, signs of AMD were identified on OCT in 1110 (6.9%) eyes where CFP were considered ungradable (847 eyes) or without signs of AMD (263 eyes). Conversely, CFP identified clinical signs of AMD in 157 (1.0%) eyes apparently free of AMD signs on OCT scans (Table 1). AMD was detected in 1789 eyes (12.4%), considering both gradable CFP and OCT images.

**Table 1:** Comparison between color fundus photography and optical coherence tomography.

		CFP assessment, no. (%)				
		No AMD (0, 1)	AMD (2, 3, 4)	Other diseases (5)	Ungradable	Total
**OCT assessment, no. (%)**	No AMD (0)	6414 (40.2%)	157 (1.0%)	171 (1.1%)	5880 (36.8%)	12622 (79.1%)
	AMD (1, 2)	263 (1.6%)	443 (2.8%)	62 (0.4%)	847 (5.3%)	1615 (10.1%)
	Other diseases (3)	96 (0.6%)	15 (0.09%)	634 (4.0%)	612 (3.8%)	1357 (8.5%)
	Ungradable	66 (0.4%)	2 (0.01%)	33 (0.2%)	262 (1.6%)	363 (2.3%)
	Total	6839 (42.9%)	617 (3.9%)	900 (5.6%)	7601 (47.6%)	15957

No. = number; AMD = age-related macular degeneration; CFP = color fundus photography; OCT = optical coherence tomography.

### Inter-operator agreement

A total of 1978 CPF and OCT images were analyzed by operator 2, too. Regarding CFP, a good inter-operator agreement was calculated for the dichotomous parameter “gradable versus ungradable” (AC1 = 0.63). A strong agreement was calculated for both parameters “presence of AMD” (AC1 = 0.96) and “AMD grading” (AC1 = 0.85) (Table 2).

**Table 2:** Inter-operator agreement on fundus photos analysis.

Parameter	Operator 1 vs. Operator 2					Gwet’s AC_1_		
	No.	− −	++	− +	+ −	AC_1_	ASE	95% CL
Gradable vs ungradable images, no. (%)	1978	705 (35.6)	960 (48.5)	31 (1.6)	282 (14.3)	0.625	0.020	0.587–0.664
Presence of AMD, no. (%)	819	741 (90.5)	45 (5.5)	26 (3.2)	7 (0.8)	0.956	0.008	0.910–0.971
Grading	819					0.853	0.014	0.852–0.881

No. = number; − = negative evaluation; + = positive evaluation; AC = agreement coefficient; ASE = asymptotic standard error; 95% CL = 95% confidence limits. AMD = age-related macular degeneration.

As regards OCT assessment, a strong inter-operator agreement was calculated for all parameters: “gradable versus ungradable” (AC1 = 0.98), “presence of AMD” (AC1 = 0.92) and “AMD grading” (AC1 = 0.93) (Table 3).

**Table 3:** Inter-operator agreement on OCT analysis.

Parameter	Operator 1 vs Operator 2					Gwet’s AC1		
	No.	− −	++	− +	+ −	AC_1_	ASE	95% CL
Gradable vs ungradable images, no. (%)	1978	33 1.7%	1916 96.9%	19 0.9%	10 0.5%	0.984	0.003	0.976–0.990
Presence of AMD, no. (%)	1737	1527 88%	108 6.2%	96 5.5%	6 0.3%	0.917	0.008	0.901–0.934
Grading	1737					0.929	0.007	0.916–0.942

No. = number; − = negative evaluation; + = positive evaluation; AC = agreement coefficient; ASE = asymptotic standard error; 95% CL = 95% confidence limits; OCT = optical coherence tomography; AMD = age-related macular degeneration.

## Discussion

AMD may cause significant visual impairment due to the involvement of the macular region, up to the development of the total loss of central vision, even though the initial stages of the disease may be completely asymptomatic [3]. Due to the important impact on patients' quality of life, the relevant economic burden and the limited availability of long term effective therapeutic options, the relevance of an effective screening protocol for detecting and grading AMD is mandatory [1,2,17–20]. CFP has been the gold standard for detecting early and late AMD, and has been shown to have a high sensitivity and specificity compared to fundus examination [7,8,21]. However, a main issue of this imaging modality is image quality, particularly when non mydriatic cameras are used in large screening settings. Keel et al., in order to develop a deep-learning algorithm for detecting wet AMD, analyzed 56113 fundus photos, and discarded 44.3% of them, considered ungradable [22]. We also found that in 47.6% of eyes CFP were ungradable for detecting and grading AMD. CFP grading may be probably improved by pupil dilatation, which, however, requires a specialized ophthalmic outpatient setting, limiting the number of the screened population. Therefore, we analyzed the individual value and the possible contribution of OCT to CFP in the screening of AMD in a general, unselected population. This approach has already been suggested, but still not extensively quantified [23]. Leuschen et al, evaluating Age-Related Eye Disease Study 2 (AREDS2) patients with OCT, reported only 2% of ungradable images [24]. In our study, OCT provided gradable images in a significantly greater number of cases compared to CFP: 15594 out of 15957 versus 8356 out of 15957, respectively. This discrepancy may be due to the fact that the acquisition of CFP is more affected by extrinsic factors than OCT, mainly due to the technologic limitations of the procedure. The better compliance of OCT versus CFP in older people may also be considered. Le Tien et al. already reported the relevance of age in obtaining gradable non mydriatic CFP: in their study, hospitalized patients older than 70 years allowed gradable CFP in 43% of eyes compared to 90.9% of eyes of a younger group of asymptomatic patients, older than 55 years [25]. De Bats et al. studied the role of non-mydriatic CFP as a screening tool for AMD, analyzing 1363 images. Compared to our results, the Authors reported only 20% of ungradable CFP [26]. This difference may be justified by the exclusion, by De Bats et al, of all non-collaborative patients and the smaller sample size. Zapata et al also reported a small percentage of ungradable CFP (586 of 50384: 1.2%) obtained during a screening program, but the Authors refer this result to the images selection performed by the examiners [27].

In our screening setting, OCT provided gradable images in 96.6% of eyes ungradable at CFP, confirming the general data of 97.7% of gradable OCT images in the whole population. Thus, adding a single linear high-resolution OCT scan through the fovea proved to significantly improve AMD detection and grading compared to CFP alone in a general, unselected population. More precisely, 847 eyes classified as ungradable by CFP (5.3% of total images) had AMD signs at OCT. OCT has already proved to detect AMD features in a significant number of eyes overlooked by CFP, but not yet in a screening setting [28]. Moreover, in our study, AMD was also detected, by OCT, in 263 eyes, classified without AMD by CFP (1.6% of total eyes). Therefore, 6.9% of the total sample was diagnosed as AMD affected eye by OCT alone.

The presence of AMD among our gradable eyes (12.4%) was substantially similar to that reported by the most recent metanalysis involving European people (range:12.3–18.5%). A higher rate of late AMD was found in our population (1.8% vs 0.50–0.75%), due to OCT, not yet used in the majority of epidemiologic AMD studies [1,2,29]. These data confirm previous studies, as the one by Leuschen et al, that found signs of advanced AMD at OCT in 16.8% of 313 eyes scored as intermediate AMD at CFP [24]. The grading system used in this study does not include a further characterization of AMD grades, (i.e. geographic atrophy versus fibrotic scar in late AMD, type and location of neovascularization). However, this kind of analysis was beyond the aim of this study, that was specifically designed for a screening setting, to identify, with a high sensitivity, patients requiring a subsequent referral for specialistic management.

One limitation of our approach may be the analysis of a single OCT scan, not identifying lesions located outside this slab. This approach may have limited the diagnostic yield offered by a full scan OCT [28]. Notwithstanding, shortening the time of examination may contribute to patient’s compliance and better fixation in a large screening setting, improving the quality of OCT imaging. Moreover, the simultaneous recording of CFP and high quality OCT may also contribute to shorten the time of image acquisition. According to our results, the addition of this OCT scan is able to significantly increase the screening power of CFP, moreover it involves the more relevant area of the macula, both in terms of frequency of disease involvement and functional relevance. In this study, CFP added to linear OCT is important to allow the visualization of the entire macular area, detecting some AMD cases undetectable at OCT (157 of 15957 cases: 1%). The ability of OCT and CFP to detect and quantify different AMD features, such as small and large drusen and hyperpigmentation, has already been reported [9,30]. Therefore, even if OCT is superior to CFP in AMD detection, as clearly demonstrated by our data, the two techniques may be defined complementary [7,9,30].

In the future, with the standardization and validation of deep learning software, the evaluation of more than one imaging modality in the same eye will contribute to an integrated multimodal imaging classification to improve screening protocols of major retinal binding conditions, such as AMD [8,31]. Moreover, both CFP and OCT have already proved to be appropriate for telemedicine-based screening and management [7,26,32]. To validate our approach, the inter-operator reproducibility was also assessed in a relevant number of images. The results clearly show the high level of reproducibility of both CFP and OCT scans in AMD screening, confirming the value for future software-based approaches.

This study confirms that the screening of AMD should be based at least on the complementary use of two imaging modalities: CFP and OCT. The use of OCT in the screening of AMD should be considered mandatory and currently complementary to CFP due to its high yield, reduced costs and simple accessibility. Further studies are necessary to evaluate the best OCT scan pattern and a more detailed score for AMD screening.
